# Ironing out the wrinkles and folds in the epidemiology of skin fold dermatitis in dog breeds in the UK

**DOI:** 10.1038/s41598-022-14483-5

**Published:** 2022-07-06

**Authors:** Dan G. O’NeillI, Dara Rowe, Dave C. Brodbelt, Camilla Pegram, Anke Hendricks

**Affiliations:** 1 Pathobiology and Population Sciences, The Royal Veterinary College, Hatfield, Herts UK; 2 Clinical Science and Services, The Royal Veterinary College, Hatfield, Herts UK

**Keywords:** Evolution, Anatomy, Diseases, Medical research, Risk factors, Signs and symptoms

## Abstract

Skin fold dermatitis (intertrigo) is an inflammatory process of closely apposing skin surfaces. Extreme conformations towards folded skin in many dog breeds are linked with higher risk. Using anonymised primary-care veterinary data from the VetCompass Programme, this study aimed to report the frequency, demographic risk factors and clinical management for skin fold dermatitis in the UK. Risk factor analysis used random effects multivariable logistic regression modelling. From a study population of 905,553 dogs, the one-year period prevalence in dogs overall was 0.37% (95% CI 0.35–0.39). Diagnosis was supported by laboratory testing in 4.21% cases. Systemic antibiosis was used in 42.30% cases. Compared with crossbreed dogs, the most highly predisposed breeds were English Bulldog (odds ratio [OR] 49.07, 95% CI 37.79–63.70), French Bulldog (OR 25.92, 95% CI 19.62–34.26,) and Pug (OR 16.27, 95% CI 12.20–21.69). The most protected breeds were Yorkshire Terrier (OR 0.14, 95% CI 0.03–0.56), Border Collie (OR 0.31, 95% CI 0.11–0.84), Jack Russell Terrier (OR 0.53, 95% CI 0.30–0.92) and Labrador Retriever (OR 0.57, 95% CI 0.35–0.93). This study adds further evidence to the welfare concerns around high popularity of dog breeds with extreme conformations. The three breeds with by far the highest odds of skin fold dermatitis represent an extreme brachycephalic conformation.

## Introduction

Skin fold dermatitis (intertrigo, intertriginous dermatitis, frictional dermatitis) is understood clinically as a superficial inflammatory process of closely apposing skin surfaces resulting from abrasion through friction, excessive moisture and reduced ventilation^1,2^. In humans, skin fold dermatitis is considered as a multi-factorial problem involving individual and environmental factors^3^ that create conditions conducive to microbial overgrowth, and bacterial and fungal skin infections^2,4^. Skin fold dermatitis and its microbial complications are also recognised in the dog and are thought to follow a similar pathogenesis to humans^5,6^. Cutaneous inflammation may result where folding of skin occurs due to deliberately selected or unplanned conformational features, or subsequent to skin thickening related to obesity or skin disease^7^. The clinical severity of skin fold dermatitis can range from mild inflammation to deep and painful ulceration^5,8^. Lesions tend to be most severe where accumulation of secretions within the folds promotes further maceration and microbial growth^6^.

Typical locations for skin fold dermatitis in dogs include facial folds of many brachycephalic breeds (e.g. French Bulldog), lower lips in breeds with large lip flaps (e.g. English Cocker Spaniel), peri-vulval in obese females with infantile vulva, around corkscrew tails (e.g. English Bulldog), in the neck fold in dogs with dewlaps (e.g. Basset Hound), between the rows of mammary glands where these are prominent, around the scrotum, as well as on the body and limbs in obese dogs or those with breed-related folding of the skin (e.g. Dachshund)^6,7^. However, not every dog with skin folds necessarily shows clinically relevant skin fold dermatitis, suggesting that additional factors such as the extent and duration of skin folding, aging and comorbidity with other generalised skin disorders may be involved^7^. Diagnosis of skin fold dermatitis is based on clinical examination supported by sampling to identify microbial complications and to direct appropriate therapy^5^. Currently proposed treatment principles for skin fold dermatitis focus on removal of surface microbes, other debris and secretions, as well as resolving microbial infection and controlling moisture, usually with topical antimicrobial and anti-inflammatory care^7^. Where present, it is also recommended to address obesity or underlying skin disease such as demodicosis^5,6^. Proactive, ongoing and long-term treatment is often required where longer term skin apposition cannot be eliminated^5,7^. Surgical intervention to remove the apposition of skin surfaces may be required where medical management is ineffective or unsustainable^6^.

Skin fold dermatitis is a commonly recognised problem in veterinary practice^5^, and was the third most commonly recorded diagnosis in the English Bulldog (8% of dogs affected)^9^, a breed with conformational predisposition to facial and tail fold dermatitis^6,8,10^. The Universities Federation for Animal Welfare (UFAW) assessed the welfare impact of facial skin fold dermatitis as a moderately severe problem due to its life-long irritation with higher welfare consequences for predisposed breeds that show more severe episodes of pain and infection^11^. In consequence, many sources of detailed online advice are presented for owners of affected breeds by animal charities^12,13^, online veterinary health advice sites^14^, the UK Kennel Club^15^, and specific breed–related sources^16^ that emphasise the welfare harms from skin fold disease and advocate skin care protocols for treatment and prevention. With dramatically rising worldwide popularity of some brachycephalic breeds over the past decade, skin fold dermatitis is receiving increased attention^17^ as evidenced by the large number of online instructive videos focusing on facial and tail fold care in brachycephalic breeds^18^. However, despite this interest in clinical care for affected dogs, there remains a deficiency of robust epidemiological evidence on skin fold dermatitis and its management practices in the wider dog population.

Using anonymised veterinary clinical data from the VetCompass Programme^19^, this study aimed to report the frequency and risk factors for skin fold dermatitis in dogs in the UK, with particular focus on identifying breed and body conformation associations. The study also aimed to report on the most undertaken clinical management strategies in the UK veterinary primary care setting. These results could assist veterinary practitioners, welfare scientists, breeders, and owners with a stronger evidence base to predict, prevent, and better manage skin fold dermatitis in dogs.

## Results

### Prevalence

**Figure 1:**
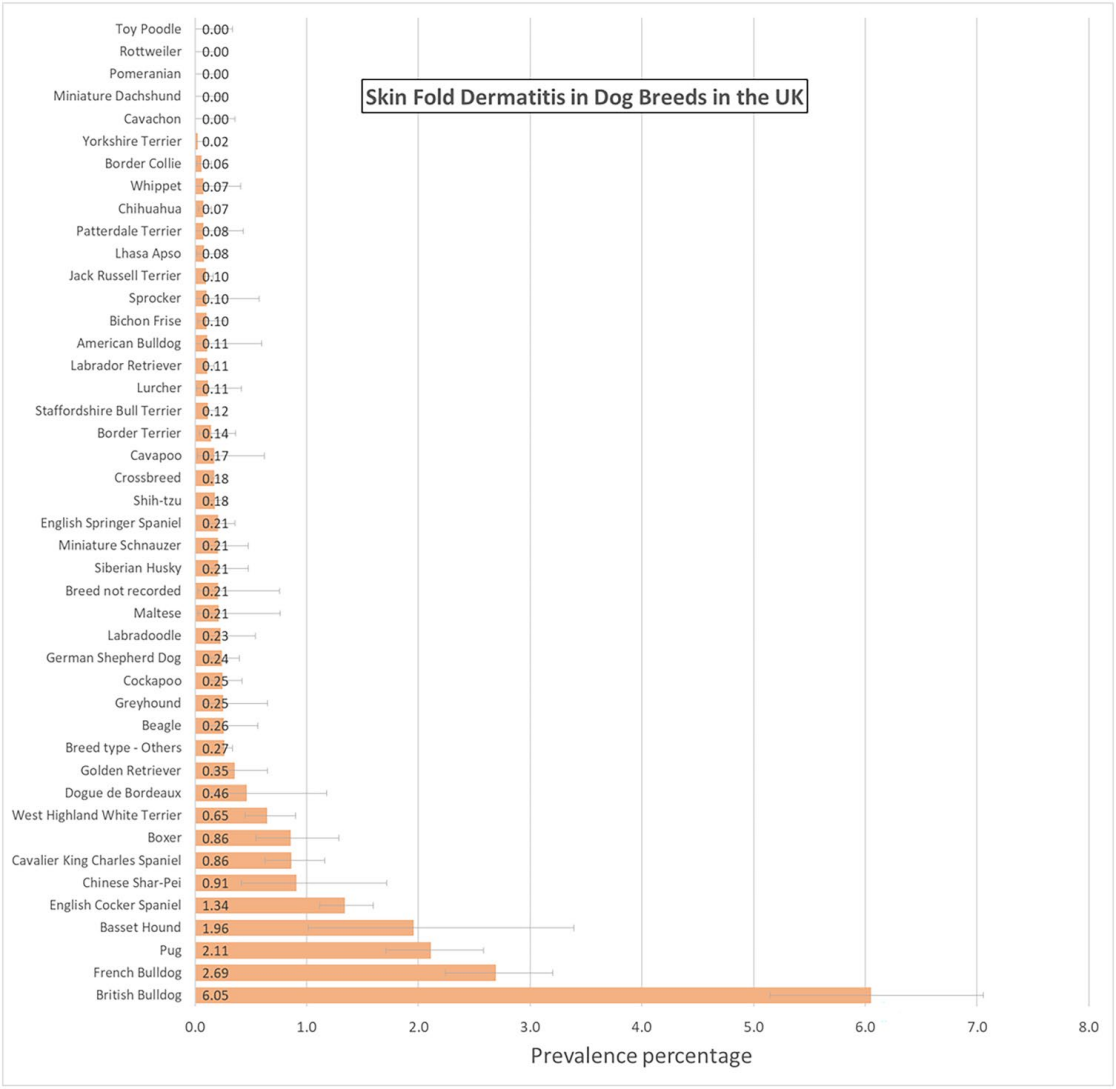
One-year (2016) period prevalence (percentage) of skin fold dermatitis in dog breeds under primary veterinary care in the VetCompass Programme in the UK. The horizontal bars represent 95% confidence intervals.

Text searches of the overall study population of 905,553 dogs under veterinary care in 2016 at 887 veterinary clinics yielded 11,375 candidate skin fold dermatitis cases. Manual checking of a random sample of 3307 (29.07%) candidate cases identified 974 confirmed skin fold dermatitis cases during 2016. After accounting for the subsampling protocol, the estimated one-year period prevalence for skin fold dermatitis in dogs overall was 0.37% (95% CI 0.35–0.39). Breeds with the highest annual prevalence of skin fold dermatitis were English Bulldog (prevalence 6.05%, 95% CI 5.15–7.06), French Bulldog (2.69%, 95% CI 2.24–3.20), Pug (2.11%, 95% CI 1.71–2.58), Basset Hound (1.96%, 95% CI 1.01–3.39) and English Cocker Spaniel (1.34%, 95% CI 1.12–1.59). Five breeds did not have any cases of skin fold dermatitis recorded: Cavachon, Miniature Dachshund, Pomeranian, Rottweiler, Toy Poodle (Fig. 1). The prevalence of skin fold dermatitis in brachycephalic breeds overall was 1.02% (95% CI 0.94–1.12) compared with a prevalence of 0.26% (95% CI 0.23–0.29) in mesocephalic breeds and 0.22% (95%CI 0.16–0.30) in dolichocephalic breeds.

Of the skin fold dermatitis cases with data available for that variable, 841 (86.52%) were purebred, 493 (50.72%) were female and 513 (52.78%) were neutered. Dogs with skin fold dermatitis had a median adult bodyweight of 14.85 kg (IQR: 10.45–24.72, range 1.10–73.05) and median age was 5.10 years (IQR: 2.29–8.45, range 0.27–17.25). The most common breeds among the skin fold dermatitis cases were English Bulldog (n = 151, 15.50%), English Cocker Spaniel (127, 13.04%), French Bulldog (123, 12.63%), Crossbreed (99, 10.16%) and Pug (94, 9.65%) (Table 1).

**Table 1:** Descriptive and univariable logistic regression results for breed as a risk factor for skin fold dermatitis during 2016 in dogs under primary veterinary care in the VetCompass Programme in the UK.

Breed	Case No (%)	Non-case No. (%)	Odds ratio	95% CI	Category *P*-value	Variable *P*-value
Crossbreed	99 (10.16)	193,388 (21.63)	Base			< 0.001
English Bulldog	151 (15.50)	8063 (0.90)	36.58	28.36–47.18	< 0.001	
French Bulldog	123 (12.63)	15,291 (1.71)	15.71	12.05–20.48	< 0.001	
Pug	94 (9.65)	14,973 (1.67)	12.26	9.24–16.27	< 0.001	
Basset Hound	12 (1.23)	2070 (0.23)	11.32	6.21–20.65	< 0.001	
English Cocker Spaniel	127 (13.04)	32,135 (3.59)	7.72	5.93–10.04	< 0.001	
Chinese Shar-Pei	9 (0.92)	3386 (0.38)	5.19	2.62–10.28	< 0.001	
Cavalier King Charles Spaniel	43 (4.41)	16,954 (1.90)	4.95	3.46–7.09	< 0.001	
Boxer	23 (2.36)	9122 (1.02)	4.93	3.13–7.76	< 0.001	
West Highland White Terrier	35 (3.59)	18,510 (2.07)	3.69	2.51–5.43	< 0.001	
Dogue de Bordeaux	4 (0.41)	2963 (0.33)	2.64	0.97–7.17	0.057	
Golden Retriever	10 (1.03)	9667 (1.08)	2.02	1.05–3.87	0.034	
Breed – Others	74 (7.60)	95,632 (10.70)	1.51	1.12–2.04	0.007	
Beagle	6 (0.62)	7961 (0.89)	1.47	0.65–3.36	0.358	
Greyhound	4 (0.41)	5422 (0.61)	1.44	0.53–3.92	0.474	
Cockapoo	13 (1.33)	18,129 (2.03)	1.40	0.79–2.50	0.253	
German Shepherd Dog	15 (1.54)	21,163 (2.37)	1.38	0.80–2.38	0.240	
Labradoodle	5 (0.51)	7419 (0.83)	1.32	0.54–3.23	0.549	
Maltese	2 (0.21)	3231 (0.36)	1.21	0.30–4.90	0.790	
Breed not recorded	2 (0.21)	3271 (0.37)	1.19	0.29–4.84	0.804	
Siberian Husky	5 (0.51)	8330 (0.93)	1.17	0.48–2.88	0.729	
Miniature Schnauzer	5 (0.51)	8335 (0.93)	1.17	0.48–2.88	0.729	
English Springer Spaniel	12 (1.23)	20,021 (2.24)	1.17	0.64–2.13	0.606	
Shih-tzu	17 (1.75)	32,578 (3.64)	1.02	0.61–1.71	0.942	
Cavapoo	2 (0.21)	4000 (0.45)	0.98	0.24–3.96	0.974	
Border Terrier	4 (0.41)	9600 (1.07)	0.81	0.30–2.21	0.686	
Staffordshire Bull Terrier	18 (1.85)	52,757 (5.90)	0.67	0.40–1.10	0.113	
Lurcher	2 (0.21)	6005 (0.67)	0.65	0.16–2.64	0.547	
Labrador Retriever	19 (1.95)	59,522 (6.66)	0.62	0.38–1.02	0.059	
American Bulldog	1 (0.10)	3184 (0.36)	0.61	0.09–4.40	0.627	
Bichon Frise	4 (0.41)	13,174 (1.47)	0.59	0.22–1.61	0.306	
Sprocker	1 (0.10)	3321 (0.37)	0.59	0.08–4.22	0.598	
Jack Russell Terrier	14 (1.44)	48,365 (5.41)	0.57	0.32–0.99	0.046	
Lhasa Apso	3 (0.31)	12,481 (1.40)	0.47	0.15–1.48	0.197	
Patterdale Terrier	1 (0.10)	4434 (0.50)	0.44	0.06–3.16	0.415	
Chihuahua	8 (0.82)	36,708 (4.11)	0.43	0.21–0.88	0.020	
Whippet	1 (0.10)	4673 (0.52)	0.42	0.06–3.00	0.386	
Border Collie	4 (0.41)	24,295 (2.72)	0.32	0.12–0.87	0.026	
Yorkshire Terrier	2 (0.21)	28,097 (3.14)	0.14	0.03–0.56	0.006	
Cavachon	0 (0.00)	3517 (0.39)	~			
Miniature Dachshund	0 (0.00)	4799 (0.54)	~			
Pomeranian	0 (0.00)	6210 (0.69)	~			
Rottweiler	0 (0.00)	7258 (0.81)	~			
Toy Poodle	0 (0.00)	3754 (0.42)	~			

Column percentages shown in brackets.

*CI* confidence interval.

Of the dogs that were not skin fold dermatitis cases with data available on the variable, 645,188 (72.42%) were purebred and 426,073 (47.88%) were female, 402,181 (45.19%) were neutered. The median adult bodyweight for non-cases was 13.93 kg (IQR: 8.15–25.00, range 0.72–97.20) and the median age was 4.44 years (IQR: 1.87–8.08, range 0.00–20.97). The most common breeds among the non-case dogs were crossbred (n = 193,388, 21.63%), Labrador Retriever (59,522, 6.66%), Staffordshire Bull Terrier (52,757, 5.90%), Jack Russell Terrier (48,365, 5.41%) and Chihuahua (36,708, 4.11%) (Table 1).

### Clinical

There were 1009 locations of skin fold dermatitis recorded among the 974 cases: 3 locations of skin fold dermatitis were recorded in 1 (0.10%) dog, 2 locations in 34 (3.49%) dogs and a single location in the remaining 939 (96.41%) dogs. Of the 974 dogs, the most common locations for skin fold dermatitis as recorded in the clinical records were lip (n = 358, 36.76%), facial fold (214, 21.97%), vulva (138, 14.17%), nasal fold (88, 9.03%), tail (56, 5.75%) and periocular fold (34, 3.49%) (Table 2).

**Table 2:** Commonly recorded locations for skin fold dermatitis in the 10 breeds with the highest prevalence in dogs under primary veterinary care during 2016 in the VetCompass Programme in the UK.

Breed	Case no	Facial (generalized or location not specified) no. (%)	Nasal no. (%)	Periocular no. (%)	Lips no. (%)	Tail no. (%)	Vulval no. (%)	Remainder no. (%)
English Bulldog	151	68 (45.03)	29 (19.21)	6 (3.97)	2 (1.32)	34 (22.52)	3 (1.99)	21 (13.91)
English Cocker Spaniel	127	3 (2.36)	0 (0)	0 (0)	118 (92.91)	0 (0)	5 (3.94)	2 (1.57)
French Bulldog	123	70 (56.91)	9 (7.32)	8 (6.5)	6 (4.88)	14 (11.38)	11 (8.94)	13 (10.57)
Pug	94	41 (43.62)	39 (41.49)	4 (4.26)	1 (1.06)	0 (0)	3 (3.19)	8 (8.51)
Cavalier King Charles Spaniel	43	0 (0)	0 (0)	4 (9.3)	33 (76.74)	0 (0)	4 (9.3)	5 (11.63)
West Highland White Terrier	35	0 (0)	0 (0)	0 (0)	27 (77.14)	0 (0)	10 (28.57)	0 (0)
Boxer	23	8 (34.78)	2 (8.7)	2 (8.7)	8 (34.78)	0 (0)	1 (4.35)	4 (17.39)
Basset Hound	12	0 (0)	0 (0)	0 (0)	4 (33.33)	0 (0)	1 (8.33)	8 (66.67)
Chinese Shar-Pei	9	2 (22.22)	0 (0)	0 (0)	1 (11.11)	1 (11.11)	0 (0)	6 (66.67)
Dogue de Bordeaux	4	1 (25)	0 (0)	0 (0)	0 (0)	1 (25)	0 (0)	2 (50)
Remainder	353	21 (5.95)	9 (2.55)	10 (2.83)	158 (44.76)	6 (1.7)	100 (28.33)	52 (14.73)
Total	974	214 (21.97)	88 (9.03)	34 (3.49)	358 (36.76)	56 (5.75)	138 (14.17)	121 (12.42)

The frequency of recorded locations for skin fold dermatitis differed widely between the breeds with high prevalence (Table 2). Three breeds with extreme brachycephaly (English Bulldog, French Bulldog and Pug) showed high levels of facial, nasal, and periocular locations. In contrast, the lips were the dominant location in the spaniel breeds (English Cocker Spaniel and Cavalier King Charles Spaniel) and the West Highland White Terrier.

No specific clinical signs were recorded in 424 (43.53%) cases. In the remaining 550 cases, erythema was recorded in 189 (34.36%) cases, inflammation in 133 (24.18%) cases, moistness in 113 (20.55%), malodour in 102 (18.55%) cases and pain in 99 (18.00%) cases.

Laboratory testing was not recorded as supporting the diagnosis of skin fold dermatitis in 933/974 (95.79%) cases. From the 41 dogs with at least one laboratory test reported, the most common laboratory tests were bacterial culture and sensitivity (n = 18, 1.85% of total), skin scraping (12, 1.23%), tape strip (6, 0.62%) and swab with cytology (6, 0.62%).

The most used medical treatments to treat skin fold dermatitis in the 974 cases were systemic antibiosis (n = 412, 42.30%), antibacterial ± antifungal shampoo/cleanser (382, 39.22%), antibacterial ± antifungal ± glucocorticoid creams/ointment (296, 30.39%), antibacterial ± antifungal wipes (151, 15.50%) and systemic glucocorticoids (132, 13.55%). Surgical management was undertaken in 15 (1.54%) of cases while 2 (0.21%) of cases were referred for advanced clinical management.

### Risk factors

**Figure 2:**
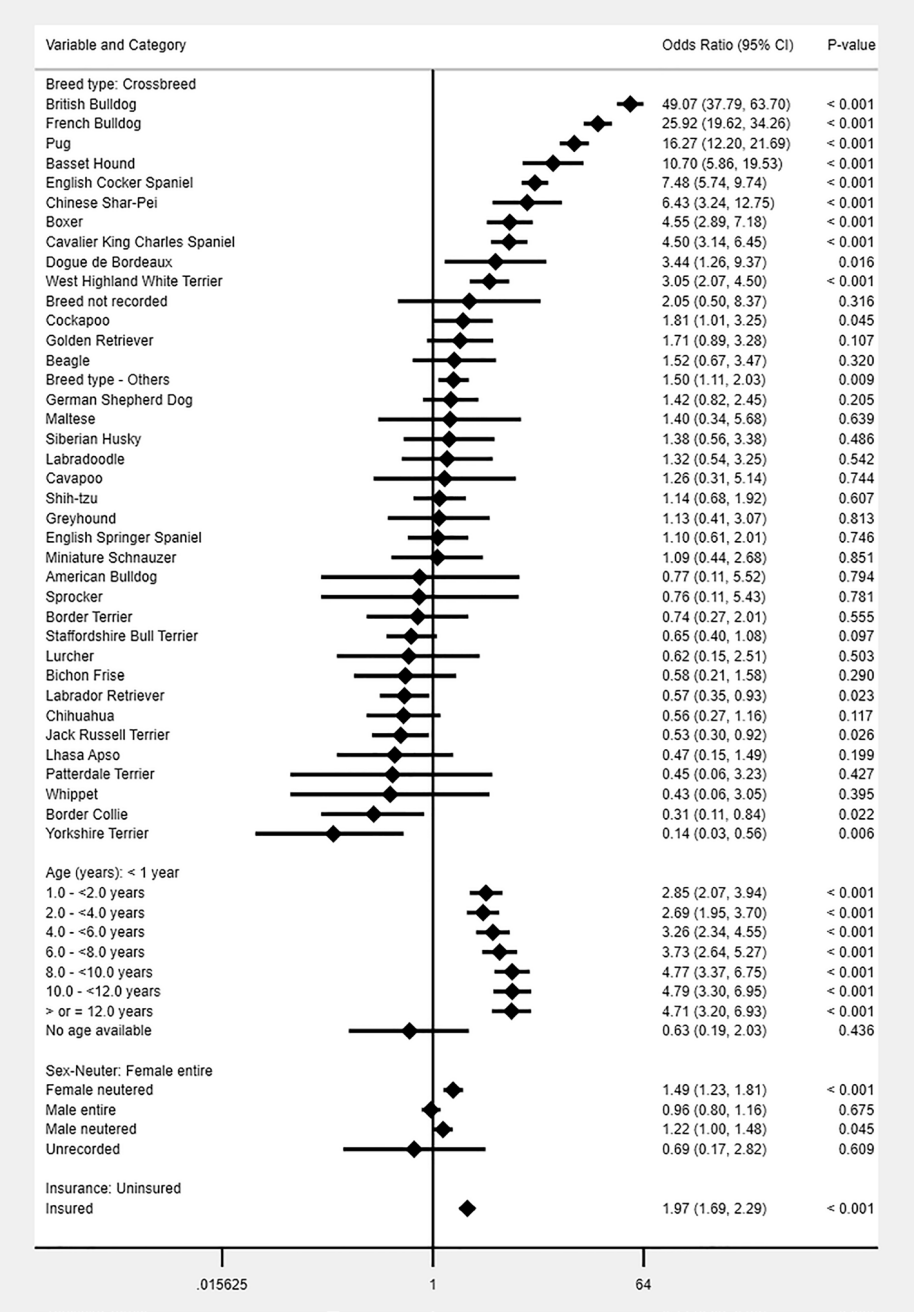
Final breed-focused mixed effects multivariable logistic regression model for risk factors associated with skin fold dermatitis in dogs under primary veterinary care in the VetCompass Programme in the UK. Clinic attended was included as a random effect. *CI confidence interval.

All study variables were liberally associated with skin fold dermatitis in univariable logistic regression modelling and were evaluated using multivariable logistic regression modelling (Tables 1, 3 and 4). The final breed-focused multivariable model retained five risk factors: *breed, age, sex-neuter* and *insurance* (Fig. 2). *Bodyweight relative to breed-sex mean* was not associated with the odds of skin fold dermatitis and was not retained in the final model. No biologically significant interactions were identified. The final model was improved by inclusion of the clinic attended as a random effect (rho: 0.05 indicating that 5% of the variability was accounted for by the clinic attended, *P* < 0.001). The final model showed acceptable model-fit (Hosmer–Lemeshow test statistic: *P* = 0.232) and acceptable discrimination (area under the ROC curve: 0.833).

**Table 3:** Descriptive and univariable logistic regression results for breed-derived risk factors for skin fold dermatitis during 2016 in dogs under primary veterinary care in the VetCompass Programme in the UK.

Variable	Category	Case No. (%)	Non-case No. (%)	Odds ratio	95% CI	Category *P*-value	Variable *P*-value
Breed purity	Crossbred	99 (10.19)	193,388 (21.71)	Base			< 0.001
	Designer	32 (3.29)	52,321 (5.87)	1.19	0.80–1.78	0.382	
	Purebred	841 (86.52)	645,188 (72.42)	2.55	2.07–3.14	< 0.001	
Kennel Club Recognised Breed	Not recognised	143 (14.71)	260,542 (29.24)	Base			< 0.001
	Recognised	829 (85.29)	630,355 (70.76)	2.40	2.01–2.86	< 0.001	
Kennel Club Breed Group	Not Kennel Club recognised breed	143 (14.71)	260,542 (29.24)	Base			< 0.001
	Terrier	81 (8.33)	144,862 (16.26)	1.02	0.78–1.34	0.894	
	Gundog	180 (18.52)	133,805 (15.02)	2.45	1.97–3.05	< 0.001	
	Working	40 (4.12)	38,628 (4.34)	1.89	1.33–2.68	< 0.001	
	Pastoral	23 (2.37)	52,615 (5.91)	0.80	0.51–1.24	0.311	
	Utility	317 (32.61)	99,302 (11.15)	5.82	4.77–7.09	< 0.001	
	Hound	27 (2.78)	31,060 (3.49)	1.58	1.05–2.39	0.028	
	Toy	161 (16.56)	130,083 (14.60)	2.26	1.80–2.82	< 0.001	
Haircoat length	Long	39 (4.00)	91,373 (10.22)	Base			< 0.001
	Medium	272 (27.93)	189,370 (21.18)	3.37	2.41–4.71	< 0.001	
	Short	523 (53.70)	333,387 (37.28)	3.68	2.65–5.09	< 0.001	
	Uncategorised	140 (14.37)	280,038 (31.32)	1.17	0.82–1.67	0.383	
Skull conformation	Mesocephalic	314 (32.24)	414,192 (46.32)	Base			< 0.001
	Brachycephalic	482 (49.49)	161,799 (18.09)	3.93	3.41–4.53	< 0.001	
	Dolichocephalic	45 (4.62)	69,197 (7.74)	0.86	0.63–1.17	0.336	
	Uncategorised	133 (13.66)	248,980 (27.84)	0.71	0.58–0.86	0.001	
Spaniel	Non spaniel-type	651 (66.84)	569,777 (63.72)	Base			< 0.001
	Spaniel-type	190 (19.51)	75,411 (8.43)	2.21	1.88–2.59	< 0.001	
	Uncategorised	133 (13.66)	248,980 (27.84)	0.47	0.39–0.56	< 0.001	

Column percentages shown in brackets.

*CI* confidence interval.

**Table 4:** Descriptive and univariable logistic regression results for non-breed-related demographic risk factors evaluated for skin fold dermatitis during 2016 in dogs under primary veterinary care in the VetCompass Programme in the UK.

Variable	Category	Case No. (%)	Non-case No. (%)	Odds ratio	95% CI	Category *P*-value	Variable *P*-value
Adult (> 18 months) bodyweight (kg)	< 10.0	165 (16.94)	211,174 (23.62)	Base			< 0.001
	10.0–< 15.0	219 (22.48)	96,237 (10.76)	2.91	2.38–3.57	< 0.001	
	15.0–< 20.0	106 (10.88)	68,308 (7.64)	1.99	1.56–2.54	< 0.001	
	20.0–< 25.0	84 (8.62)	62,909 (7.04)	1.71	1.31–2.22	< 0.001	
	25.0–< 30.0	86 (8.83)	52,857 (5.91)	2.08	1.60–2.70	< 0.001	
	30.0–< 40.0	80 (8.21)	68,882 (7.70)	1.49	1.14–1.94	0.004	
	≥ 40.0	20 (2.05)	25,855 (2.89)	0.99	0.62–1.57	0.966	
	Uncategorised	214 (21.97)	307,946 (34.44)	0.89	0.73–1.09	0.258	
Bodyweight relative to breed mean	Lower	368 (37.78)	313,161 (35.02)	Base			< 0.001
	Equal/Higher	390 (40.04)	270,939 (30.30)	1.22	1.06–1.41	0.005	
	Uncategorised	216 (22.18)	310,068 (34.68)	0.59	0.50–0.70	< 0.001	
Age (years)	< 1.0	49 (5.03)	103,020 (11.52)	Base			< 0.001
	1.0–< 2.0	162 (16.63)	128,751 (14.40)	2.65	1.92–3.64	< 0.001	
	2.0–< 4.0	193 (19.82)	175,810 (19.66)	2.31	1.69–3.16	< 0.001	
	4.0–< 6.0	157 (16.12)	138,202 (15.46)	2.39	1.73–3.29	< 0.001	
	6.0–< 8.0	128 (13.14)	111,884 (12.51)	2.41	1.73–3.34	< 0.001	
	8.0–< 10.0	128 (13.14)	89,787 (10.04)	3.00	2.16–4.17	< 0.001	
	10.0–< 12.0	84 (8.62)	65,327 (7.31)	2.70	1.90–3.85	< 0.001	
	≥ 12.0	70 (7.19)	69,019 (7.72)	2.13	1.48–3.07	< 0.001	
	Uncategorised	3 (0.31)	12,368 (1.38)	0.51	0.16–1.64	0.258	
Sex	Female	493 (50.62)	426,073 (47.65)	Base			0.082
	Male	479 (49.18)	463,890 (51.88)	0.89	0.79–1.01	0.076	
	Uncategorised	2 (0.21)	4,205 (0.47)	0.41	0.10–1.65	0.210	
Neuter	Entire	459 (47.13)	487,783 (54.55)	Base			< 0.001
	Neutered	513 (52.67)	402,181 (44.98)	1.36	1.20–1.54	< 0.001	
	Uncategorised	2 (0.21)	4,204 (0.47)	0.51	0.13–2.03	0.336	
Insurance	Non-insured	725 (74.44)	779,462 (87.17)	Base			< 0.001
	Insured	249 (25.56)	114,706 (12.83)	2.33	2.02–2.70	< 0.001	

Column percentages shown in brackets.

*CI* confidence interval.

After accounting for the effects of the other variables evaluated, 11 breeds showed increased odds of skin fold dermatitis compared with crossbred dogs. Breeds with the highest odds included English Bulldog (OR 49.07, 95% CI 37.79–63.70), French Bulldog (OR 25.92, 95% CI 19.62–34.26), Pug (OR 16.27, 95% CI 12.20–21.69) and Basset Hound (OR 10.70, 95% CI 5.86–19.53). Four breeds showed reduced odds of skin fold dermatitis compared with crossbreds: Labrador Retriever (OR 0.57, 95% CI 0.35–0.93), Jack Russell Terrier (OR 0.53, 95% CI 0.30–0.92), Border Collie (OR 0.31, 95% CI 0.11–0.84) and Yorkshire Terrier (OR 0.14, 95% CI 0.03–0.56). Five breeds had no recorded skin fold dermatitis cases. All older age groups had higher odds compared with dogs aged under one year, with odds rising as dogs aged. Neutered animals had higher odds than entire animals within both sexes. Insured dogs had 1.97 (95% CI 1.69–2.29) times the odds of skin fold dermatitis compared with uninsured dogs (Fig. 2).

**Figure 3:**
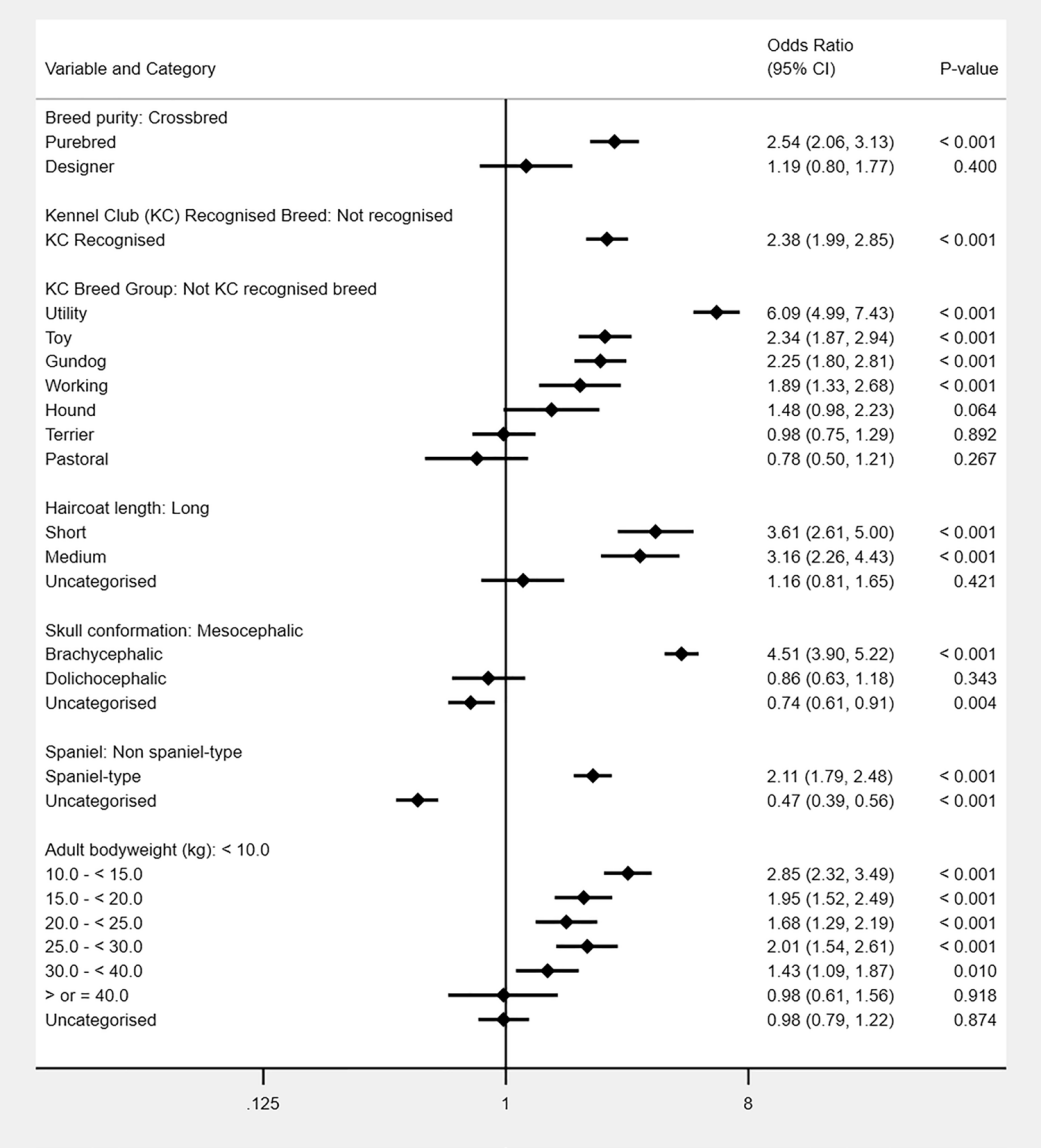
Results for risk factors that directly replaced the breed variable in the final breed-focused mixed effects multivariable logistic regression model (along with age, sex/neuter and insurance status). These results report associations between these risk factors and skin fold dermatitis in dogs under primary veterinary care in the VetCompass Programme in the UK. Clinic attended was included as a random effect. *CI confidence interval.

As described in the methods, breed-derived variables were introduced individually to replace *breed* in the final breed-focused model. Compared with crossbred dogs, purebred dogs had increased odds (OR 2.54, 95% CI 2.06–3.13) of skin fold dermatitis. Four Kennel Club breed groups showed higher odds compared to breeds that were not recognised by the Kennel Club: utility, toy, gundog and working. Compared with breeds with long coats, breeds with short (OR 3.61, 95% CI 2.61–5.00) and medium (OR 3.16, 95% CI 2.26–4.43) coats showed increased odds of skin fold dermatitis. Compared with breeds with mesocephalic skull conformation, breeds with brachycephalic skull conformation (OR 4.51, 95% CI 3.90–5.22) had increased odds of skin fold dermatitis. Spaniel types had 2.11 times the odds (95% CI 1.79–2.48) of skin fold dermatitis compared with non-spaniel types. Dogs with an adult bodyweight under 10 kg had lower odds compared with dogs weighing from 10 to 40 kg (Fig. 3).

## Discussion

This study estimates a one-year prevalence of 0.37% for skin fold dermatitis in the general population of dogs under primary veterinary care in the UK. The study also highlights several breeds with high prevalence values. The wider canine welfare relevance from skin fold dermatitis arises from the high (and rising) popularity of some of these commonly affected breeds, with four of the five most frequently affected breeds also being amongst the 10 most commonly registered Kennel Club breeds in the UK^20^: French Bulldog (2.7% prevalence, Kennel Club popularity no. 2), Cocker Spaniel (1.3%, no.3), English Bulldog (6.0%, no.4) and Pug (2.1%, no.9). The breed-specific prevalence for English Bulldog, Pug and French bulldog reported in the current study concur with earlier studies that reported across all common disorders in those individual breeds^9,21,22^.

However, despite these high breed-specific prevalence values reported here, it is likely that studies based on primary-care veterinary clinical records underestimate the true frequency of skin fold dermatitis because of systematic under-recording of observed problems in electronic records, with only 64% of observed problems being recorded into the practice EPR in one study^23^. Additionally, 37% of owners of English Bulldogs with skin disease of any type in Finland failed to identify these animals as having a health problem, and so therefore many affected dogs may not even be presented for veterinary care^24^. Under-recognition by owners of skin fold dermatitis as a clinical problem may be even higher than for other types of skin disease because the abnormal skin is generally concealed deep within the skin folds. Furthermore, pernicious effects from the normalisation phenomenon of breed-related issues (i.e., ‘normal for breed’) may result in not just reduced owner recognition of skin fold dermatitis as a clinical problem but could also reduce veterinary recognition or recording^25–27^.

Ultra-predispositions have been defined as predisposition with odds over 4 times higher compared with a broad comparator group^28^. In the current study, ultra-predispositions were shown for 8 breeds: English Bulldog, French Bulldog, Pug, Basset Hound, English Cocker Spaniel, Chinese Shar-Pei, Boxer and Cavalier King Charles Spaniel. It would be logical to prioritise review of the breed standards for each of these ultra-predisposed breeds to remove any wording that encourages harmful skin folding^29^. Welfare, veterinary and breed associations could also make greater efforts to inform the wider general public that, although humans may perceive folded skin as ‘cute’, this conformation is instead a pathology that carries high welfare implications for affected breeds and individuals^27^.

The current study aimed to add information on breed protection, as well as on breed predispositions, to enable a more differentiated consideration of aetiopathogenesis. For instance, the current study highlighted contrasting frequencies of skin fold dermatitis for four breeds predisposed to atopic dermatitis but without significant skin folding^30,31^, with two breeds (West Highland White Terrier, Golden Retriever) predisposed to skin fold dermatitis while two were protected (Jack Russell Terrier, Labrador Retriever). Furthermore, breeds with both predisposed and protected statuses co-occurred within genetically close terrier and retriever clades^32^. Clearly, skin fold dermatitis is an aetiopathogenetically complex disorder, with much remaining to be learned about the aetiological pathways.

At an individual patient level, most affected dogs (96.41%) had just one location of skin fold dermatitis recorded during the study period. Whilst this may be an accurate reflection of patient status, one of the author’s (AH) referral practice experience suggests that, where skin fold dermatitis is detected in more than one location, it is often only the site of most concern that is recorded in the primary-care patient record. However, despite this limitation, the current study still identified majorly differing patterns of location between commonly affected breeds^29^. Among the three breeds with extreme brachycephalic conformations, English Bulldogs, French Bulldogs and Pugs, facial (facial, nasal and peri-ocular) locations of fold dermatitis were common; this could reflect the failure of muzzle skin to be reduced in proportion to the extreme brachycephalic facial skeleton. A high frequency of tail fold dermatitis was also recorded in English Bulldogs and French Bulldogs, two breeds that commonly show screw tails^33,34^. Conversely, high frequency of fold dermatitis in locations other than the face was shown in the Bassett Hound and Shar Pei, two breeds with breed standards that promote wide-spread wrinkling of body and extremities^29^. However, the high predilection of the lips as the location for fold dermatitis in affected Cocker Spaniels (93%) and West Highland White Terriers (77%) suggests that factors other than the extent of skin folding can contribute to this disorder because these two breeds show muzzle conformations that are broadly similar to protected breeds such as Labrador Retrievers, Jack Russell Terriers or Border Collies^29^. This opens speculation that factors promoting maceration, skin barrier damage and susceptibility to skin infection^35^, or concurrent inflammatory skin disease are relatively more important in these breeds than physical factors related to skin folding. The role of moisture as one such alternative risk factor may be represented by the Cavalier King Charles Spaniel, with 9% of cases in that breed recorded with periocular fold dermatitis. This breed has a mild brachycephalic conformation without significant facial folding^36^, but a high frequency of ocular disorders^37^. Excess tearing (epiphora) or use of eye treatments related to these ocular disorders may result in prolonged wetting of the shallow skin fold originating near the medial canthus and could promote skin maceration and consequent skin fold dermatitis.

Compared with dogs aged under one year, the odds of diagnosis with skin fold dermatitis rose as dogs aged. The biological basis of this observation is not obvious. The limited information available on the effect of physiological ageing on skin structure and function in dogs would suggest that skin thickness, measured with calipers, reduces with age but not epidermal thickness or elasticity^38,39^, and that the bacterial community structure in healthy dogs did not differ by age^40,41^. A fuller understanding of geriatric skin changes is compounded by regional, individual, breed, sex, time, and disease effects on biophysical parameters^38,40–43^. It is conceivable that chronic, if initially low grade, inflammation of skin folds results in chronic hyperplastic dermatitis characterized by dermal and epidermal thickening through oedema, inflammatory infiltrates, glandular hyperplasia, and fibrosis. The progression of these structural and functional changes over time may precipitate or exacerbate chafing, secondary infection and thus increase the odds of diagnosis with aging.

Long hair was protective from diagnosis with skin fold dermatitis in the current study. Whilst there may be an effect of hair coat type and length on friction, moisture retention or skin microbiome and thus predisposition to skin fold inflammation, at present this remains speculative. It is also possible that signs of skin fold dermatitis may be more easily detected in dogs with a short hair coat and therefore this may lead to a correspondingly higher rate of diagnosis.

The current results revealed that 96% of diagnoses of skin fold dermatitis were not accompanied by laboratory testing. This finding concurs with skin fold dermatitis being firmly regarded as a clinical diagnosis based on inflammatory skin lesions limited to the area of skin apposition^5–7^. In line with this view, where clinical signs were specifically recorded in this study, these reflected signs such as inflammation and associated malodour and pain that are detectable organoleptically. Pain was noted in nearly one in five dogs that had a record of clinical signs, underscoring the welfare relevance of skin fold dermatitis as a chronic condition. Whilst laboratory testing was uncommon in this primary-care study, this is recommended to rule out primary causes of dermatitis such as demodicosis, and to identify the presence of deeper infection or the presence and nature of the microbial overgrowth that could alter the therapeutic approach^5,6^. Although bacteria and Malassezia yeasts are considered to play a central role in skin fold dermatitis, their involvement is frequently limited to high populations on the skin surface^7^.

In the absence of a published evidence base for treatment of skin fold dermatitis in dogs, there is agreement on the general principles of treatment, if not the specific treatment modalities. Removal of surface microbial load, debris and exudation is considered a crucial treatment principle, along with resolution of tissue infection (if present), and glucocorticoid-aided control of inflammation is also deemed helpful for resolution^5,6^. For both approaches, topical treatment modalities are generally recommended. Acceptance and adoption of these principles is reflected in the current study where 84% of cases were prescribed antimicrobial ± glucocorticoid topical treatment. Lasting resolution of skin fold dermatitis is rarely achieved where friction and moisture retention continue at sites of close skin apposition, and therefore longer-term topical preventive or maintenance treatment is usually required^5–7^. Although skin barrier repair products are used for treatment and prevention of skin fold dermatitis in humans^44^, these have not yet been proposed in the veterinary literature but would seem an interesting concept to explore prospectively for prevention in predisposed dogs.

In line with responsible antimicrobial stewardship principles in dogs, systemic antibiotics are recommended for use only after confirmation of deep bacterial infection by cytology, and culture and susceptibility testing to direct drug choice^5,6^. This strict approach is in contrast with the current results, where systemic antibiosis was prescribed in over 40% of cases, whereas bacterial culture was performed in less than 2%. Extrapolating from the overall period prevalence of skin fold dermatitis and the antibiotic treatment frequency in the current study, 0.16% of dogs in primary care practice received systemic antimicrobials for treatment of skin fold dermatitis over the one-year period. While this shows that skin fold dermatitis treatment contributes significantly less to systemic antimicrobial use in canine practice compared with anal sac disorders and pyoderma, with an estimated 1% of UK dogs being treated with systemic antibiosis each year for each of these disorders^45,46^, the current results still highlight the widespread use of systemic antibiosis without documented diagnostic justification. Selection bias towards formal diagnosis of more severe presentations of skin fold dermatitis may partially explain the high frequency of systemic antimicrobial prescriptions documented in the current study that seems misaligned with the heavy focus on treating using only topical modalities in published, empirical treatment recommendations^5–7^. Assessment of disease severity, or depth of infection, was not possible in the current study and therefore fuller speculation about the clinical justification for systemic antibiotic treatment in such a large proportion of dogs was not possible.

Epidemiological studies based on primary care practice records uniquely reflect common clinical practice for the patient demographics and professional standards that are specific to the professional, economic and societal context at the time of sampling^47^. Whilst this is a strength, offering opportunities to define benchmarks and compare current practice to guidance, there are several limitations that have been previously reported to this approach of using a convenience sample of practices and to the nature of data that were not primarily recorded for research purposes and thus subject to data gaps and inaccuracies^47,48^. The diagnosis of skin fold dermatitis relies heavily on typical clinical signs evident on inspection of the skin, which should translate into a high diagnostic specificity. However as discussed above, issues around owner awareness and factors relating to thresholds for clinical diagnosis and recording may have created considerable and variable selection bias between practices and clinicians. The current study was a prevalence study that included all cases diagnosed with skin fold dermatitis in 2016 rather than an incidence study that only included cases that were first diagnosed in 2016. While this approach may give stronger inference on the disorder burden from skin fold dermatitis on dogs, it could also bias towards subsets of dogs with greater survival or earlier first diagnosis appearing to have higher odds. The statistical precision was weak for results from uncommon breeds and body sites affected, and therefore these associations should be treated with caution. Treatment information was based on medicines dispensed and may not capture recommendations of general sales items that may be purchased away from the veterinary surgery.

In conclusion, this study has explored anonymised primary-care veterinary clinical data in the UK to show that 0.37% of dogs overall were recorded with skin fold dermatitis annually. The three breeds with by far the highest odds of skin fold dermatitis represent breeds with an extreme brachycephalic conformation: English Bulldog, French Bulldog and Pug. The evident contribution of breed-related conformation for this condition adds to the discussion on the welfare and ethical challenges around current high demand for extreme conformations in dogs. The high frequency of systemic antimicrobial usage for skin fold dermatitis documented in this study can be regarded as a benchmark of current clinical activity and also raises questions around misalignment of UK primary-care prescribing practices with published treatment recommendations.

## Methods

The study population included dogs under primary veterinary care at clinics participating in the VetCompass Programme during 2016. Dogs under veterinary care were defined as those with either (a) ≥ 1 electronic patient record (EPR) (free-text clinical note, treatment, or bodyweight) recorded during 2016 or (b) ≥ 1 EPR recorded during both 2015 and 2017. VetCompass collates de-identified EPR data from primary-care veterinary practices in the UK for epidemiological research^19^. Data fields available to VetCompass researchers include a unique animal identifier along with species, breed, date of birth, sex, neuter status, insurance and bodyweight, and also clinical information from free-form text clinical notes, summary diagnosis terms^49^ and treatment with relevant dates.

A cohort study design was used to estimate the one-year (2016) period prevalence of skin fold dermatitis and to explore associations with demographic risk factors in this population. In a UK study using primary-care clinical records, skin fold dermatitis did not feature among disorders with a prevalence of 0.46% and above^50^. Therefore, assuming a prevalence lower than 0.46%, power calculations estimated that a study sample with at least 38,136 dogs was needed to estimate prevalence for a disorder that occurred in 0.25% of dogs with 0.05% acceptable margin of error at a 95% confidence level from a national UK population of 8 million dogs^51,52^. Ethics approval was obtained from the RVC Ethics and Welfare Committee (reference SR2018-1652). All methods were performed in accordance with the relevant guidelines and regulations. The study is reported in accordance with ARRIVE guidelines^53^.

The case definition for a skin fold dermatitis case required evidence in the clinical records indicating a final diagnosis of skin fold dermatitis or synonym (e.g., intertrigo, skin fold pyoderma) at any date from Jan 1, 2016 to Dec 31, 2016. Each dog was defined as either a case or non-case based on the meeting the case definition above, regardless of the level of veterinary care received during 2016. Case-finding involved initial screening of all 905,554 study dogs for candidate skin fold dermatitis cases by searching the clinical free-text from July 1st 2015 to June 30th 2017 using the search terms: wrinkl*, intertrig*, pyod* + fold*, derm* + fold*, fac* + fold*, lip* + fold*, neck* + fold*, body* + fold*, vulv* + fold*, tail* + fold*, nas* + fold*, infect* + fold*, inflam* + fold*, prurit* + fold*, bact* + fold*, yeast* + fold*, chin* + fold*. The clinical notes of a random sample of candidate animals were manually reviewed to evaluate for case inclusion. Additional information was extracted for each confirmed skin fold dermatitis case: body location of the skin fold dermatitis, clinical signs, diagnostic testing and treatment/management.

Breed descriptive information entered by the participating practices was cleaned and mapped to a VetCompass breed list derived and extended from the VeNom Coding breed list that included both recognised purebred breeds and also designer-crossbreed breed terms^49^. A *breed purity* variable categorised all dogs of recognisable breeds as ‘purebred’, dogs with contrived names generated from two or more purebred breed terms as ‘designer’ crossbreds (purposely bred crossbreeds) and dogs recorded as mixes of breeds but without a contrived name as ‘crossbred’^15^. A *breed* variable included individual pure breeds and designer hybrids represented by over 3000 dogs in the overall study population or with ≥ 5 skin fold dermatitis cases, along with groupings of all remaining breeds and also general crossbred dogs. This approach was taken to facilitate statistical power for the individual breed analyses^54^. Breeds were also characterised by haircoat (short, medium, long, uncategorised), skull shape (dolichocephalic, mesocephalic, brachycephalic, uncategorised) and spaniel (spaniel, non-spaniel, uncategorised) status for analysis. A *Kennel Club breed group* variable classified breeds recognised by the UK Kennel Club into their relevant breed groups (Gundog, Hound, Pastoral, Terrier, Toy, Utility and Working) and all remaining types were classified as non-Kennel Club recognised^15^.

Consistent with methods previously used^55^, neuter and insurance status were defined by the final available EPR value. Adult bodyweight was defined as the mean of all bodyweight (kg) values recorded for each dog after reaching 18 months old and was categorised as: < 10.0, 10.0 to < 15.0, 15.0 to < 20.0, 20.0 to < 25.0, 25.0 to < 30.0, 30.0 to < 40.0 and ≥ 40.0. Mean adult bodyweight was generated for all breed/sex combinations with adult bodyweight available for at least 100 dogs in the overall study population and used to categorise individual dogs as “at or above the breed/sex mean”, “below the breed/sex mean” and “unspecified”. Age (years) was defined based on the earliest date for diagnosis of skin fold dermatitis in the available clinical records for cases and on December 31, 2016 (the final date in 2016 that these dogs were not a case) for non-cases. Age was categorised as: ≤ 1.0, 1.0 to < 2.0, 2.0 to < 4.0, 4.0 to < 6.0, 6.0 to < 8.0, 8.0 to < 10.0, 10.0 to < 12.0 and ≥ 12.0.

Following internal validity checking and data cleaning in Excel (Microsoft Office Excel 2013, Microsoft Corp.), analyses were conducted using Stata Version 16 (Stata Corporation). The one-year period prevalence with 95% confidence intervals (CI) described the probability of skin fold dermatitis at any point during 2016. Because the sampling design involved verification of a subset of candidate cases, the predicted total case count was calculated using the Stata *survey* function as previously described^56^. The CI estimates were derived from standard errors, based on approximation to the binomial distribution^57^. Risk factor analysis used binary logistic regression modelling to evaluate univariable associations between risk factors (*breed, haircoat, skull shape, spaniel, breed purity, Kennel Club recognised breed, Kennel Club breed group, adult bodyweight, bodyweight relative to breed-sex mean, age, sex, neuter,* and *insurance*) and skin fold dermatitis during 2016. Because breed was a factor of primary interest for the study, variables that derived from the breed information and therefore were highly correlated with breed (*haircoat, skull shape, spaniel, breed purity, Kennel Club recognised breed* and *Kennel Club breed group*) were excluded from initial breed multivariable modelling. Instead, each of these variables individually replaced the *breed* variable in the main breed-focused model to evaluate their effects after taking account of the other variables. *Adult bodyweight* (a defining characteristic of individual breeds) replaced breed and *bodyweight relative to breed/sex mean* in the final breed-focused model. Risk factors with liberal associations in univariable modelling (*P* < 0.2) were taken forward for multivariable evaluation. Model development used manual backwards stepwise elimination. Clinic attended was evaluated as a random effect and pair-wise interaction effects were evaluated for the final model variables^58^. The area under the ROC curve and the Hosmer–Lemeshow test were used to evaluate the quality of the model fit and discrimination (non-random effect model)^58,59^. Statistical significance was set at *P* < 0.05.

## Data Availability

The datasets generated during and/or analysed during the current study are available at the RVC Research Online repository https://rvc.worktribe.com/record.jx?recordid=1557498.

## Abbreviations

CI: Confidence interval
EPR: Electronic patient record
IQR: Interquartile range
KC: The Kennel Club
OR: Odds ratio

## References

[1] Janniger CK, Schwartz RA, Szepietowski JC, Reich A (2005). Intertrigo and common secondary skin infections. Am. Fam. Phys..

[2] Kalra MG, Higgins KE, Kinney BS (2014). Intertrigo and secondary skin infections. Am. Fam. Physician.

[3] Gabriel S, Hahnel E, Blume-Peytavi U, Kottner J (2019). Prevalence and associated factors of intertrigo in aged nursing home residents: A multi-center cross-sectional prevalence study. BMC Geriatr..

[4] Tüzün Y, Wolf R, Engin B, Keçici AS, Kutlubay Z (2015). Bacterial infections of the folds (intertriginous areas). Clin. Dermatol..

[5] Banovic, F. & Strzok, E. Skin Fold Dermatitis (Intertrigo) in Dogs. *Today's Veterinary Practice* November/December 2019 (2019).

[6] Paterson S (2017). Intertrigo in the dog: Aetiology, clinical signs and therapy. Companion Anim..

[7] Scott, D. W., Miller, W. H. & Griffin, C. E. in *Muller & Kirk's Small Animal Dermatology* (eds D. W. Scott, W. H. Miller, & C. E. Griffin) (W.B. Saunders, 2001).

[8] Banovic F, Olivry T, Linder KE, Tobias JR (2014). Pathology in practice Psoriasiform lichenoid dermatitis. Vet. Dermatol..

[9] O’Neill DG (2019). Disorders of Bulldogs under primary veterinary care in the UK in 2013. PLoS ONE.

[10] The Kennel Club. *Breed health and conservation plans (BHCPs)*, https://www.thekennelclub.org.uk/health/breed-health-and-conservation-plans/ (2022).

[11] UFAW. *Genetic Welfare Problems of Companion Animals*, https://www.ufaw.org.uk/genetic-welfare-problems-intro/genetic-welfare-problems-of-companion-animals-intro (2022).

[12] Pavletic, M. M. *Tail Fold Intertrigo (Screw Tail)*, https://www.mspca.org/angell_services/tail-fold-intertrigo-screw-tail/ (2022).

[13] PDSA. *Skin fold dermatitis in dogs*, https://www.pdsa.org.uk/pet-help-and-advice/pet-health-hub/conditions/skin-fold-dermatitis-in-dogs (2022).

[14] Conner, D. *How to Care for Your Wrinkly Dog*, https://www.petmd.com/dog/grooming/2how-care-your-wrinkly-dog (2018).

[15] The Kennel Club. *Breed Information Centre*, https://www.thekennelclub.org.uk/search/breeds-a-to-z (2022).

[16] PetPugDog.com. *Pug Wrinkles*, http://www.petpugdog.com/pug-wrinkles (2019).

[17] Feng, T., McConnell, C., O’Hara, K., Chai, J. & Spadafori, G. Nationwide Brachycephalic Breed Disease Prevalence Study. 8 (http://nationwidedvm.com/wp-content/uploads/2017/03/NWBrachycelphalicStudy0317.pdf, 2017).

[18] The Dog Grooming Den. (2018).

[19] VetCompass. *VetCompass Programme*, http://www.rvc.ac.uk/VetCOMPASS/ (2022).

[20] Bedford, E. *Leading 20 dog breeds in the United Kingdom (UK) in 2020, based on number of registrations*. https://www.statista.com/statistics/915202/top-dog-breeds-by-registered-number-united-kingdom-uk/ (2021).

[21] O'Neill DG, Darwent EC, Church DB, Brodbelt DC (2016). Demography and health of Pugs under primary veterinary care in England. Can. Genet. Epidemiol..

[22] O'Neill DG, Baral L, Church DB, Brodbelt DC, Packer RMA (2018). Demography and disorders of the French Bulldog population under primary veterinary care in the UK in 2013. Can. Genet. Epidemiol..

[23] Jones-Diette J, Robinson N, Cobb M, Brennan M, Dean RS (2017). Accuracy of the electronic patient record in a first opinion veterinary practice. Prev. Vet. Med..

[24] Seppänen RTK (2019). Skin and ear health in a group of English bulldogs in Finland—a descriptive study with special reference to owner perceptions. Vet. Dermatol..

[25] O'Neill DG (2020). Unravelling the health status of brachycephalic dogs in the UK using multivariable analysis. Sci. Rep..

[26] Ravetz, G. *Stop normalising suffering: vets speaking out about brachys*, https://www.bva.co.uk/news-and-blog/blog-article/stop-normalising-suffering-vets-speaking-out-about-brachys/ (2017).

[27] Packer RMA, O'Neill DG, Fletcher F, Farnworth MJ (2019). Great expectations, inconvenient truths, and the paradoxes of the dog-owner relationship for owners of brachycephalic dogs. PLoS ONE.

[28] O'Neill DG (2021). French Bulldogs differ to other dogs in the UK in propensity for many common disorders: A VetCompass study. Can. Med. Genet..

[29] The Kennel Club. *Breed standards*, https://www.thekennelclub.org.uk/activities/dog-showing/breed-standards/ (2022).

[30] Jaeger K (2010). Breed and site predispositions of dogs with atopic dermatitis: A comparison of five locations in three continents. Vet. Dermatol..

[31] Mazrier H, Vogelnest LJ, Thomson PC, Taylor RM, Williamson P (2016). Canine atopic dermatitis: Breed risk in Australia and evidence for a susceptible clade. Vet. Dermatol..

[32] Parker HG (2017). Genomic analyses reveal the influence of geographic origin, migration, and hybridization on modern dog breed development. Cell Rep..

[33] Mansour TA (2018). Whole genome variant association across 100 dogs identifies a frame shift mutation in DISHEVELLED 2 which contributes to Robinow-like syndrome in Bulldogs and related screw tail dog breeds. PLoS Genet..

[34] Conte A (2021). Thoracic vertebral canal stenosis associated with vertebral arch anomalies in small brachycephalic screw-tail dog breeds. Vet. Comp. Orthop. Traumatol.

[35] Black JM (2011). MASD part 2: incontinence-associated dermatitis and intertriginous dermatitis: A consensus. J. Wound Ostomy Continence Nurs..

[36] Packer RM, Hendricks A, Burn CC (2015). Impact of facial conformation on canine health: Corneal ulceration. PLoS ONE.

[37] Summers J (2015). Prevalence of disorders recorded in Cavalier King Charles Spaniels attending primary-care veterinary practices in England. Canine Genet. Epidemiol..

[38] Young LA, Dodge JC, Guest KJ, Cline JL, Kerr WW (2002). Age, breed, sex and period effects on skin biophysical parameters for dogs fed canned dog food. J. Nutr..

[39] Kimura T, Doi K (1994). Age-related changes in skin color and histologic features of hairless descendants of Mexican hairless dogs. Am. J. Vet. Res..

[40] Torres S (2017). Diverse bacterial communities exist on canine skin and are impacted by cohabitation and time. PeerJ.

[41] Rodrigues Hoffmann A (2014). The skin microbiome in healthy and allergic dogs. PLoS ONE.

[42] Hightower K, Marsella R, Flynn-Lurie A (2010). Effects of age and allergen exposure on transepidermal water loss in a house dust mite-sensitized beagle model of atopic dermatitis. Vet. Dermatol..

[43] Oh W-S, Oh T-H (2010). Mapping of the dog skin based on biophysical measurements. Vet. Dermatol..

[44] Mistiaen P, van Halm-Walters M (2010). Prevention and treatment of intertrigo in large skin folds of adults: a systematic review. BMC Nurs..

[45] Summers JF, Hendricks A, Brodbelt DC (2014). Prescribing practices of primary-care veterinary practitioners in dogs diagnosed with bacterial pyoderma. BMC Vet. Res..

[46] O’Neill DG (2021). Non-neoplastic anal sac disorders in UK dogs: Epidemiology and management aspects of a research-neglected syndrome. Vet. Rec..

[47] O'Neill D, Church D, McGreevy P, Thomson P, Brodbelt D (2014). Approaches to canine health surveillance. Canine Genet. Epidemiol..

[48] O'Neill D, Lee MM, Brodbelt DC, Church DB, Sanchez RF (2017). Corneal ulcerative disease in dogs under primary veterinary care in England: epidemiology and clinical management. Canine Genet. Epidemiol..

[49] The VeNom Coding Group. *VeNom Veterinary Nomenclature*, http://venomcoding.org (2022).

[50] O'Neill DG, James H, Brodbelt DC, Church DB, Pegram C (2021). Prevalence of commonly diagnosed disorders in UK dogs under primary veterinary care: results and applications. BMC Vet. Res..

[51] Epi Info CDC. *Centers for Disease Control and Prevention (US): Epi Info*, https://www.cdc.gov/epiinfo/index.html (2021).

[52] Asher L (2011). Estimation of the number and demographics of companion dogs in the UK. BMC Vet. Res..

[53] ARRIVE. *ARRIVE guidelines: Animal Research: Reporting of In Vivo Experiments*, https://arriveguidelines.org/ (2022).

[54] Scott M, Flaherty D, Currall J (2012). Statistics: how many?. J. Small Anim. Pract..

[55] O'Neill DG (2021). Reporting the epidemiology of aural haematoma in dogs and proposing a novel aetiopathogenetic pathway. Sci. Rep..

[56] O'Neill DG (2016). Epidemiology of hyperadrenocorticism among 210,824 dogs attending primary-care veterinary practices in the UK from 2009 to 2014. J. Small Anim. Pract..

[57] Kirkwood, B. R. & Sterne, J. A. C. *Essential Medical Statistics*. 2nd edn, (Blackwell Science, 2003).

[58] Dohoo, I., Martin, W. & Stryhn, H. *Veterinary epidemiologic research*. 2nd edn, (VER Inc, 2009).

[59] Hosmer, D. W., Lemeshow, S. & Sturdivant, R. X. *Applied logistic regression*. 3rd edn, (Wiley, 2013).

